# Conditional Mutant *MAPT* Overexpression in Neural Organoids Elicits Robust Tau Accumulation and Pathological Transcriptomic Responses

**DOI:** 10.1021/acschemneuro.6c00376

**Published:** 2026-09-28

**Authors:** Andrew Kjar, Hyosung Kim, Madeline R. Spetz, Zev Jarrett, Melanie Fernandez, Jaime Wang, Maria L. Russotti, Caroline Bodnya, Daniel Chavarria, Timothy J. Hohman, Vivian Gama, Brad Grueter, Jonathan M. Brunger, Ethan S. Lippmann

**Affiliations:** † Department of Biomedical Engineering, 5718 Vanderbilt University, Nashville 37235-1631, Tennessee, United States; ‡ Department of Chemical and Biomolecular Engineering, Vanderbilt University, Nashville 37235-1631, Tennessee, United States; § Department of Pharmacology, Vanderbilt University, Nashville 37235-1631, Tennessee, United States; ∥ Vanderbilt Memory and Alzheimer’s Center, 12328 Vanderbilt University Medical Center, Nashville 37232-2102, Tennessee, United States; ⊥ Department of Cell and Developmental Biology, 12327 Vanderbilt University School of Medicine, Nashville 37232, Tennessee, United States; # Vanderbilt Brain Institute, Vanderbilt University, Nashville 37235-1631, Tennessee, United States; ∇ Vanderbilt Center for Stem Cell Biology, Vanderbilt University, Nashville 37235-1631, Tennessee, United States; ○ Department of Anesthesiology, Vanderbilt University Medical Center, Nashville 37235-1631, Tennessee, United States; ◆ Vanderbilt Center for Addiction Research, Vanderbilt University, Nashville 37235-1631, Tennessee, United States; ¶ Vanderbilt-Ingram Cancer Center, Vanderbilt University Medical Center, Nashville 37232-2102, Tennessee, United States

**Keywords:** Tau (MAPT), neural organoids, induced pluripotent stem cells (iPSCs), neurodegeneration, disease modeling, inducible expression

## Abstract

Modeling neurodegenerative conditions in neural organoids presents a potential complement to existing preclinical translation pipelines. Prior neural organoid models have generally relied on human induced pluripotent stem cell (iPSC) lines containing disease-relevant alleles. However, this strategy limits temporal control over the initiation and progression of pathological events. In this study, we provide a proof-of-concept framework showing that neurodegenerative phenotypes can be triggered by inducible expression cassettes. We developed an iPSC line harboring doxycycline-inducible mutant *MAPT*, which encodes the microtubule-associated protein tau that is implicated in tauopathies. Induction of mutant *MAPT* via doxycycline treatment in differentiating neural organoids yielded accumulation of phosphorylated tau, which localized inside individual neuron somas within the cortical plate-like regions. Importantly, measured transcriptomic responses to tau overexpression bore key similarities to human clinical transcriptomic data. Taken together, we present an approach for controlling the initiation and progression of proteinopathy-like pathology in neural organoid systems.

## Graphical abstract

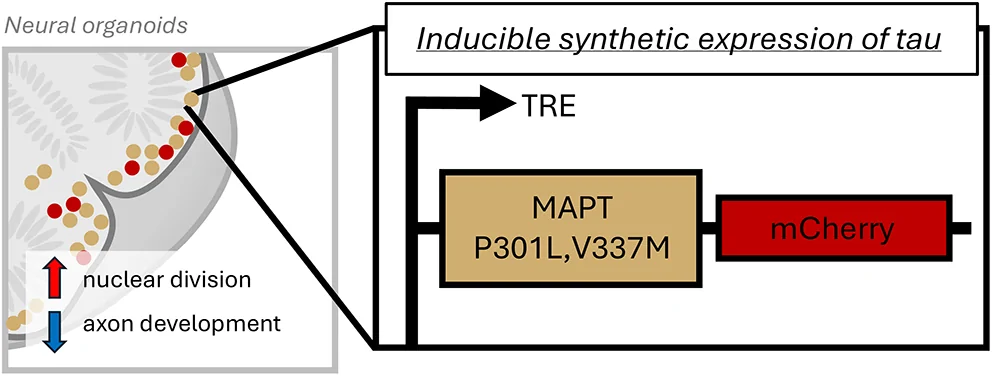

## Introduction

Tauopathies are a heterogeneous class of diseases arising from the pathological accumulation of the microtubule-associated protein tau (encoded by the *MAPT* gene).^1^ Correlated pathological signatures include loss of neuronal identity and upregulation of cell cycle transcripts in mature neurons,^2^ depletion of neuronal populations,^3^ and cognitive decline.^4^ As such, tauopathies and related dementias, including Alzheimer’s disease, are leading causes of end-of-life pathology in the United States.^5^ Due to the longstanding challenges in developing effective therapies for neurodegenerative conditions, there is an urgent need for establishing representative and translationally relevant preclinical models.^6^ As rodents do not natively develop tauopathies, a wide array of approaches to induce pathology have been implemented, including genetic manipulation or direct injections of detrimental tau aggregates (as has been extensively reviewed elsewhere^7–11^ ). Nonrodent animal models have also been employed, to varying degrees of success.^12–14^ However, it is widely recognized that differences between humans and other species can hamper the translation of findings from animal models, which motivates the development and use of complementary, human-based approaches.

Spurred on by the widespread availability of induced pluripotent stem cells (iPSCs), human cell-based model systems have received significant interest for their potential contribution to translational research.^15^ Of these systems, organoids, which are self-patterning three-dimensional tissue structures, have the advantage of diverse cell type representation and the partial recapitulation of tissue-level morphology.^16^ Organoid systems have been widely applied to modeling genetic disease, as patient-derived cells often display pathological signatures that can be detected early within organoid differentiation protocols.^17^ Moreover, they can be directly manipulated through conventional and synthetic biological techniques to harbor phenotypes of interest, study the interactions of genetic knockdowns, or understand the biological mechanisms of disease.

Building and benchmarking next-generation human cell-based tauopathy models is an ongoing goal of the research community. Previous neural organoid models of tauopathy have mostly been constructed by using iPSCs derived from familial patients harboring point mutations in key genes (ex. *APOE* or *MAPT*) or iPSCs where these key genes have been edited to include the disease-relevant mutations. In these models, accumulation of tau is the most commonly reported outcome.^18–20^ Downstream pathologies have also been reported; for example, V337M MAPT mutant iPSC lines form organoids that display gradual neuronal loss and dysregulated cholesterol biosynthesis.^21,22^ As a second approach, exogenous stimulation with patient-derived tau causes tau aggregation and neuroinflammation and can be used to model tauopathy in organoid models.^23^ Similarly, organoids exposed to human serum accumulate amyloid β in addition to tau and display impaired synapses.^24^ These models showcase the utility of organoid models in interrogating the mechanisms of tauopathy.

Systems for inducible proteinopathies, including optogenetic and doxycycline-inducible cassettes, are often implemented in monolayer cultures to model neurodegeneration^25–29^ and offer a promising third alternative. As with patient-derived systems or exogenous seeding of pathology, inducible systems can be used to assay the responses of neural cells and glia to disease, within their cellular milieu. A few notable pioneering studies have begun to implement these systems in neural organoids: for example, overexpression of mutant α-synuclein or Progerin produces Parkinson’s disease like-states in neural organoid systems,^30,31^ overexpression of TDP-43 can cause neural toxicity,^32^ and most relevantly, AAV-mediated overexpression of tau leads to tau aggregates which are immunoreactive for pathology-related antibodies (MC-1, AT8).^33^ While these studies strongly suggest that inducible systems are compatible with disease modeling in organoids, they have not yet exploited the full potential of these systems, including induction of pathology in particular subsets of cells, fine control of temporal progression of disease, or modeling of disease comorbidities. Before exploring these possibilities, a stable and convenient method for the induction of tauopathy is needed. Thus, in this work we assessed the ability of inducible expression cassette to impart tauopathy phenotypes in a neural organoid.

We provide a proof-of-concept framework showing that tauopathy phenotypes can be imparted through inducible expression of disease-associated genes. To our knowledge, our work represents the first stable integration of an inducible cassette for controlled overexpression of mutant *MAPT* in neural organoid cultures. We specifically deployed an inducible cassette which expresses full-length human tau, with two point mutations that hinder normal tau function and thereby promote aggregation (P301L and V337M). Upon transgene induction, the resultant neural organoids display robust hyperphosphorylated tau accumulation in neurons and pathogenic transcriptomic alterations. We further noted drawbacks which might be addressed in future work including difficulties in post day 10 temporal control of the cassette and relatively weak correlation to patient pathological transcriptional signatures. Taken together, these results represent progress toward modeling tau pathology in neural organoids with greater control.

## Results and Discussion

### Cell Line Engineering and Construct Validation

To implement inducible tau expression, we cloned a transgenic construct containing full length tau and mCherry separated by a P2A cleavage sequence, all under the control of the drug-inducible tetracycline response element (TRE) promoter. We built the construct with the aggregation-prone, frontotemporal dementia-related point mutations P301L and V337M layered together in the tau sequence (Figure 1A, Supporting Figure 1). These point mutations have previously been reported to disrupt physiological tau binding, thus promoting its toxic aggregation.^34^ This combination of mutations has been widely used in HEK293T cellular models and is known to readily aggregate in the presence of tau seeds.^35–37^ Although this combination of mutations is not commonly present in human populations, we reasoned that for proof-of-concept work, the use of two mutations would ensure a maximized pathological outcome. We also specifically used the 4R isoform of tau, as exon 10 (the fourth repeat domain) harbors the aggregation-prone point mutation P301L and the 4R tau isoform is involved in a variety of tauopathies, including frontotemporal dementia and Alzheimer’s disease.^38^ It is also notable that 4R isoform expression levels are typically poor in unmodified neural organoids,^21^ unlike the mature human brain, further motivating our approach.

**Figure 1:**
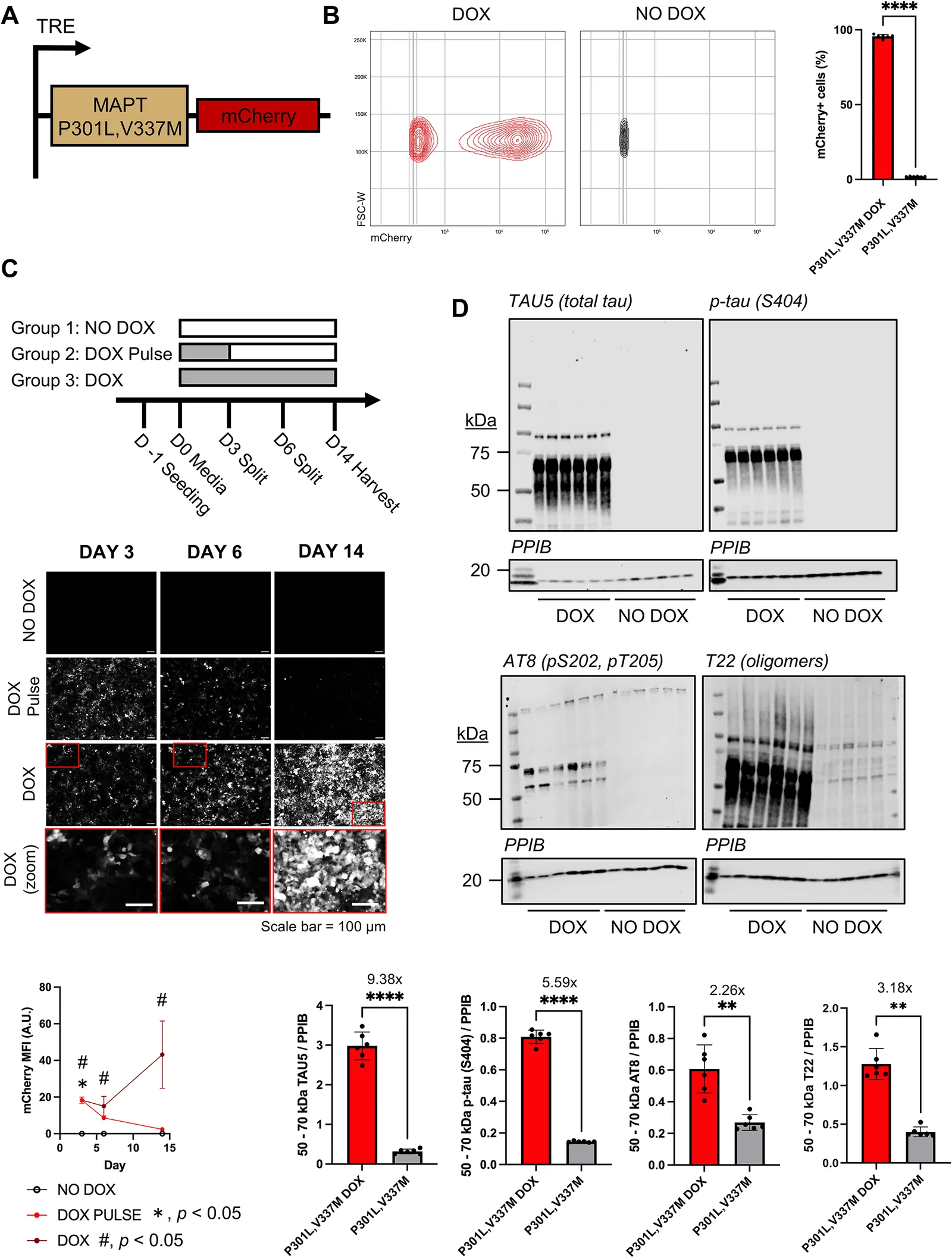
Establishment of an inducible mutant *MAPT* cassette in HEK293 cells. (A) Schematic of the inducible cassette. TRE – tetracycline response element. (B) Left: density plots of mCherry expression in engineered HEK293 cells with and without doxycycline. *Y*-axis: forward scatter width (FSC-W). Right: quantification of percent mCherry+ cells (*n* = 6 wells, technical replicates), ****p < 0.0001, Welch’s *t* test. Bars represent the mean ± standard deviation. (C) Left: schematic of experimental protocol. Center: representative live epifluorescence imaging of HEK293 monolayers at days 3, 6, and 14. Scale bar = 100 μm. Right: quantification of mCherry mean fluorescence intensity (MFI) (*n* = 3 wells per time point, technical replicates), points represent mean, bars represent standard deviation, *p < 0.05 for DOX PULSE compared to NO DOX condition, ^#^ *p* < 0.05 for DOX compared to NO DOX condition, day 3: ordinary one-way ANOVA and Holm-Sidak multiple comparisons test; day 6 and 14: Kruskal–Wallis and Dunn’s multiple comparison test. (D) Top: Western blots of cell lysates, targeted with antibodies TAU5,pS404, AT8, and T22, or PPIB (housekeeping). Bottom: quantification of 50–70 kDa TAU5/PPIB ratio and quantification of 50–70 kDa pS404/PPIB ratio (*n* = 6 wells, technical replicates), ****p < 0.0001, **p < 0.01, Welch’s *t* test (TAU5, pS404, AT8) or Mann–Whitney test (T22). Bars represent the mean ± standard deviation. Fold change is noted.

The dual mutant construct was initially transfected into HEK293 cells to test induction efficiency. The Sleeping Beauty 100x transposase system^39^ and puromycin selection were used to generate stably integrated lines. As expected, mCherry fluorescence was readily induced upon doxycycline addition, as observed by flow cytometry (Figure 1B). Fluorescence levels were reduced upon removal of doxycycline, demonstrating the responsiveness of the system (Figure 1C). At the protein level, both total tau (as measured with the TAU-5 antibody) and phosphorylated tau (pS404) signatures were readily detectable, as determined via Western blot 3 days after doxycycline induction (Figure 1D). Tau signatures related to pathology – hyperphosphorylated tau^40^ (AT8 antibody) and oligomeric tau^41^ (T22 antibody) – were also detectable due to tau overexpression. These results suggest that inducible mutant tau overexpression can yield pathological signatures.

To transition this approach into neural organoids, KOLF2.1J iPSCs^42^ were transfected with the same construct. After initial integration, we utilized fluorescence-activated cell sorting to select for top mCherry expressing iPSCs after 24 h of doxycycline treatment and then expanded the cell line in basal media conditions without doxycycline. As in HEK293 cells, sorted iPSCs expressed mCherry both two and 3 days after doxycycline addition, detectable via immunofluorescence or flow cytometry, respectively (Figure 2A–B). Without the addition of doxycycline, no mCherry was detectable, indicating the selectivity of the induction cassette. We noted heterogeneity in the population, as not all sorted cells maintained the ability to respond to doxycycline after population recovery (∼40% of cells expressed mCherry, as assayed by flow cytometry, Figure 2B). High-resolution epifluorescence imaging revealed phosphorylate tau buildup in the cell bodies of iPSCs which had been stimulated by doxycycline for 48 h, selectively in the engineered cell line (Figure 2C). Tau signal was largely dispersed, consistent with strong overexpression but not late-stage aggregate-like pathology. Western blot analysis of the protein lysates from these cells confirmed increased levels of tau and phosphorylated tau as a function of cassette induction (Figure 2D). Elevated immunoreactivity for hyperphosphorylated and oligomeric tau antibodies was also detectable, though much weaker than in the HEK293 line (Figure 2E). We also noted variability in loading control (PPIB) levels across the iPSC samples, potentially indicating some increased cellular heterogeneity due to tau construct expression. However, as the experimental focus was tau expression, all analyses were normalized to housekeeping protein levels.

**Figure 2:**
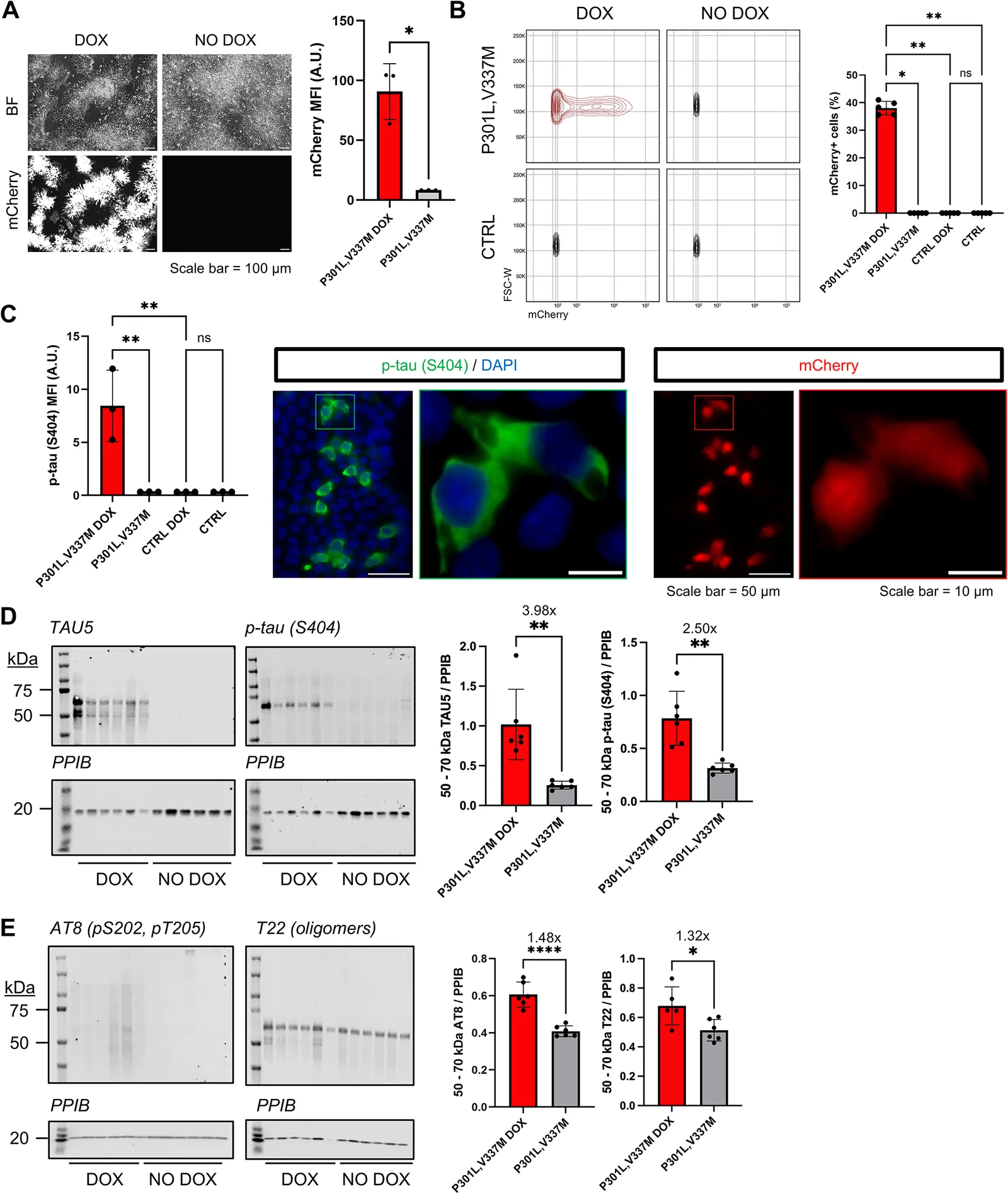
Establishment of an inducible mutant *MAPT* cassette in KOLF2.1J induced pluripotent stem cells. (A) Left: representative live brightfield (BF) and epifluorescence imaging of monolayer iPSCs, 48 h with or without doxycycline stimulation. Scale bar = 100 μm. Right: quantification of mCherry mean fluorescence intensity (MFI) (*n* = 3 wells, technical replicates). *p < 0.05, Welch’s *t* test. Bars represent the mean ± standard deviation. (B) Left: density plot of engineered iPSCs (P301L,V337M) or control iPSCs (CTRL), 72 h with or without doxycycline stimulation. *Y*-axis: forward scatter width (FSC-W). Right: quantification of percent mCherry+ cells (*n* = 5 wells, technical replicates), **p < 0.01, *p < 0.05 Kruskal–Wallis and Dunn’s multiple comparison tests. Bars represent the mean ± standard deviation. (C) Left: quantification of p-tau mean fluorescence intensity (MFI) after 48 h with or without doxycycline stimulation (*n* – 3 wells, technical replicates). **p < 0.01, ordinary one-way ANOVA, with Holm-Sidak multiple comparison tests. Bar represent the mean ± standard deviation. Right: representative epifluorescence imaging of monolayer P301L,V337 M iPSCs, 48 h with doxycycline stimulation. Scale bar = 50 μm (wider field), and 10 μm (insets). (D) Left: Western blots of cell lysates, targeted with antibodies TAU5 and pS404, or PPIB (housekeeping). Right: quantification of 50–70 kDa TAU5/PPIB ratio and quantification of 50–70 kDa pS404/PPIB ratio (*n* = 6 wells, technical replicates), **p < 0.01, Mann–Whitney test (TAU4) or Welch’s *t* test (pS404). Bars represent the mean ± standard deviation. Fold change is noted. (E) Left: Western blots of cell lysates, targeted with antibodies AT8 and T22, or PPIB (housekeeping). Right: quantification of 50–70 kDa AT8/PPIB ratio and quantification of 50–70 kDa T22/PPIB ratio (*n* = 6 wells, technical replicates), **** *p* < 0.0001, * *p* < 0.05, unpaired *t* test. Bars represent the mean ± standard deviation. Fold change is noted.

### Expression of Mutant-Tau Cassettes in Cortical Organoids

Having successfully implemented the synthetic expression cassettes in iPSCs, we next investigated the activity of these constructs in neural organoids. To generate neural organoids, we utilized a modified protocol, which includes dual-SMAD inhibition to reinforce neuroectoderm formation, and then matrix-free conditions in ultralow attachment shaking dishes for long-term maturation. Organoids were stimulated with doxycycline, which was supplemented in cell culture media starting at day 10, after initial neural patterning (Figure 3A).

**Figure 3:**
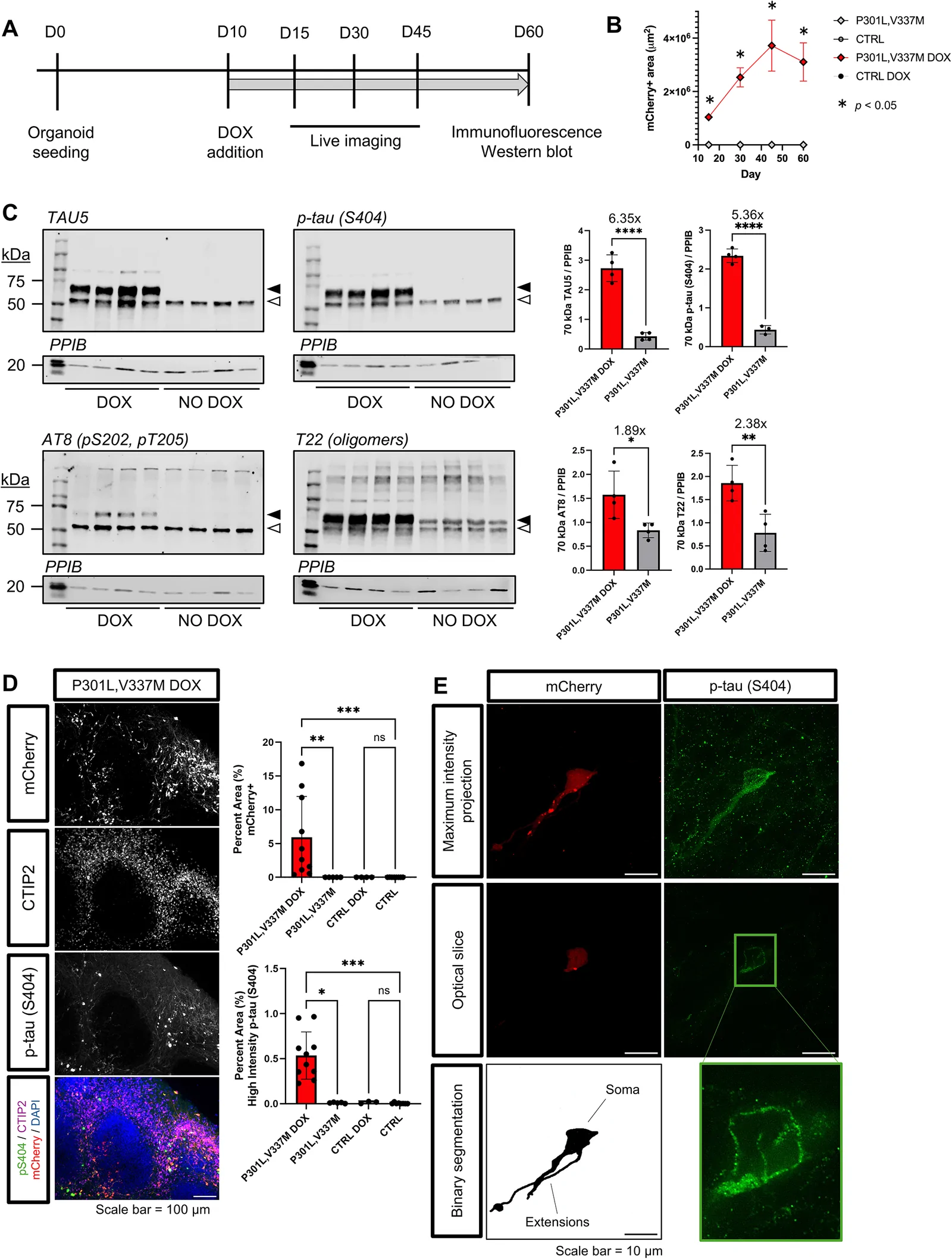
Inducible mutant *MAPT* overexpression in neural organoids yields increased levels of phosphorylated tau aggregates. (A) Schematic of experimental procedure. (B) Quantification of mCherry+ area in neural organoids, measured by live epifluorescence imaging (*n* = 3–4 individual organoids, one differentiation batch), points represent mean, bars represent standard deviation, *p < 0.05 with doxycycline compared to no doxycycline, multiple unpaired *t* tests. (C) Left: Western blots of organoid lysates (each lane represents a single organoid), targeted with antibodies TAU5, pS404, AT8, T22, or PPIB (housekeeping). Right: quantification of 50–70 kDa TAU5, pS404, AT8, T22/PPIB ratio (*n* = 3–4 individual organoids, one differentiation batch), ****p < 0.0001, **p < 0.01, *p < 0.05, unpaired *t* test. Bars represent the mean ± standard deviation. Black triangle indicates full length (4R) tau, in contrast to smaller species (3R). Fold change is noted. (D) Left: representative immunofluorescent imaging of thin-slice sections of doxycycline-treated engineered organoids, visualizing mCherry, CTIP2 (a neural marker), and pS404. Scale bar = 100 μm. Right top: quantification of percent area mCherry+ or percent area high intensity p-tau+ (pS404), (*n* = 4–10 individual organoids, two differentiation batches), ***p < 0.001, **p < 0.01, *p < 0.05, n.s. – not significant, Kruskal–Wallis and Dunn’s multiple comparisons test. Bars represent the mean ± standard deviation. Representative images of controls are shown in Supporting Figure 2. (E) Representative high-resolution confocal imaging of thin-slice section of doxycycline-treated engineered organoids, visualizing mCherry and pS404. For reference in distinguishing cell features, a labeled binary segmentation of the same image is also provided. Scale bar = 10 μm.

Live imaging of neural organoids over the first 60 days of culture confirmed mCherry expression selectively in the stimulated group (Figure 3B). Western blotting then confirmed robust tau expression, which was normalized to loading control (PPIB) expression (Figure 3C). The overexpressed tau was immunoreactive for phosphorylation signatures (S404 and AT8) and potential oligomeric species (T22). We noted that the endogenous tau bands (those detected across control conditions) were of smaller molecular weight than the induced bands, consistent with previous reports that unmanipulated neural organoids mostly express 3R instead of 4R tau,^33^ and thus also underscoring our use of full-length tau in the inducible construct design. Previous cryo-electron microscopy studies of donor tissue have shown associations with pathological folding and aggregation due to P301L or V337M mutational status.^43,44^ In future work, it will be revealing to analyze lysates from the doxycycline-treated mutant organoids, allowing direct comparison of protein conformations, as the full conformational implications of the dual mutant remain uncharacterized.

We then sought to understand the cellular distribution of the phosphorylated tau. Fixed cryosections were immunostained, imaged for phosphorylated tau (pS404), and quantified based on manual thresholds which excluded endogenous tau levels (those present in nontreated controls). This analysis, reported as high-intensity phosphorylated tau area, revealed strong tau accumulation selectively in doxycycline-treated mutant organoids (Figure 3D, representative controls shown in Supporting Figure 2). High-magnification spinning-disk confocal microscopy and image deconvolution suggested that phosphorylated tau could be detected in the soma and extensions of mCherry+ cells (Figure 3E). Across observations, phosphorylated tau was not present in cell nuclei (Supporting Figure 3).

In these experiments, a relatively small percentage (∼5%) of the resulting organoid tissue was positive for mCherry. To overcome this, we then explored if higher induction efficiency could be produced using selective pressures during organoid culture, leveraging the fact that successfully transfected cells harbor a puromycin resistant transgene in addition to the inducible mutant *MAPT* transgene. Indeed, continuous addition of puromycin to the cell culture media increased the overall amounts of mCherry+ and pS404+ cells (Figure 4A–D). As expected, the majority of mCherry+ cells display expression of p-tau (Figure 4E). However, the relative percentage of mCherry+ pS404- cells was increased as a result of puromycin treatment (p = 0.0005, two-tailed *t* test), potentially due to the underlying heterogeneity of the underlying iPSC population.

**Figure 4:**
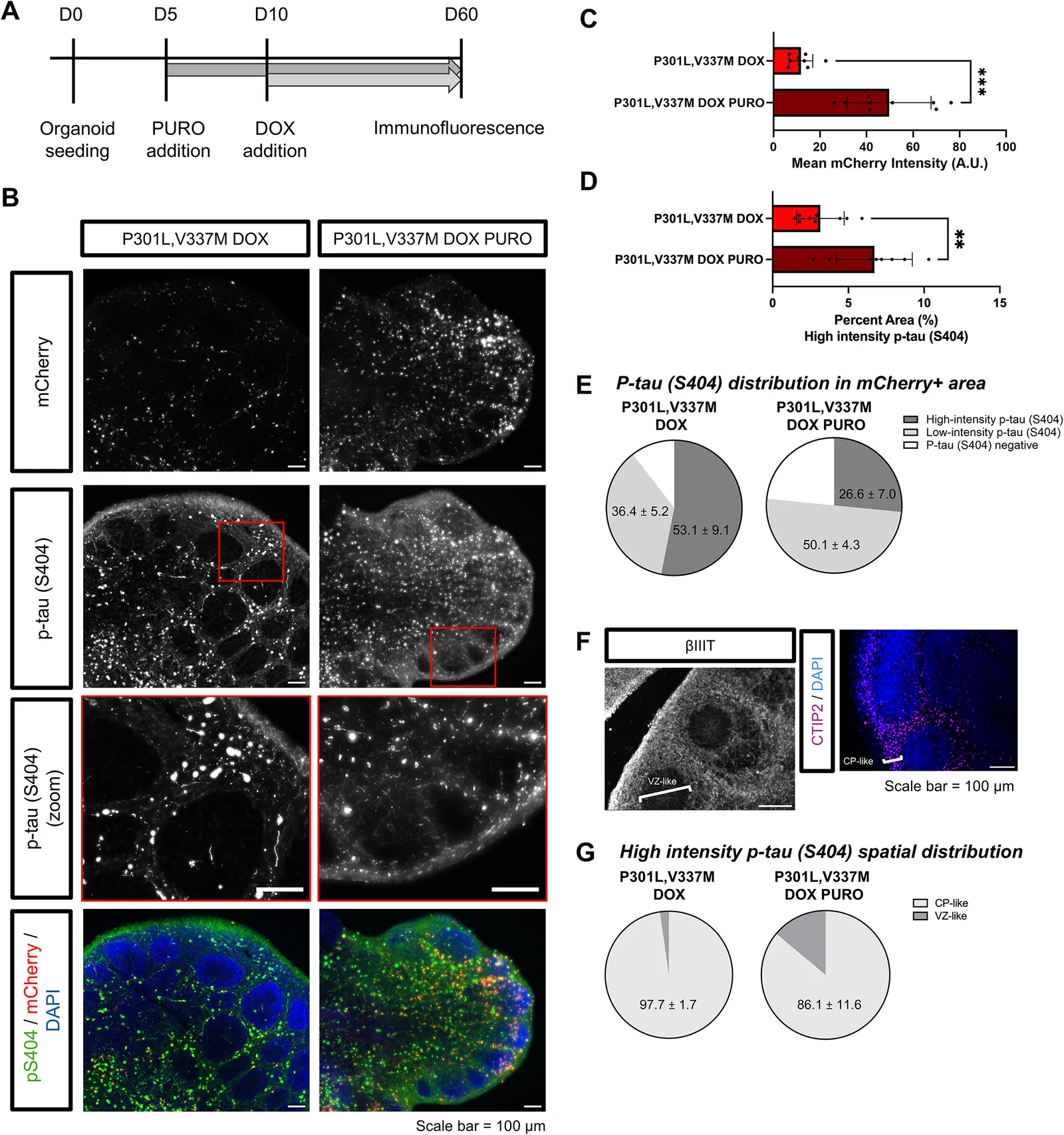
Puromycin selection during neural organoid culture improves cassette induction efficiency. (A) Schematic of experimental procedure. (B) Representative immunofluorescent imaging of thin-slice sections of doxycycline-treated (DOX) engineered organoids with and without puromycin selection (PURO), visualizing mCherry, pS404, and DAPI. Scale bar = 100 μm. (C) Quantification of percent area mCherry+, (*n* = 8–9 individual organoids, one differentiation batch), ***p < 0.001, Welch’s *t* test. Bars represent the mean ± standard deviation. (D) Quantification of percent area high intensity p-tau+ (pS404), (*n* = 8–9 individual organoids, one differentiation batch), **p < 0.01, unpaired *t* test. Bars represent the mean ± standard deviation. (E) Quantification of percent of high-intensity or low-intensity pS404 signal within mCherry+ tissue cells (*n* = 8–9 individual organoids, one differentiation batch). Mean high-intensity p-tau and low-intensity p-tau proportions is displayed ± standard deviation. (F) Representative immunofluorescent imaging of thin-slice sections of organoids, visualizing β III tubulin (BIIIT), DAPI, and CTIP2, with sections marked as ventricular zone like (VZ-like) or cortical plate like (CP-like). Scale bar = 100 μm. (G) Quantification of percent of high-intensity pS404 signal which is localized to either the CP-like (light gray) or VZ-like regions (dark gray) (*n* = 8–9 individual organoids, one differentiation batch). Mean CP-like proportion is displayed ± standard deviation.

Newer donor vector designs that resist transgene silencing could further improve this approach.^45^ This is particularly important because we also determined that initiation of doxycycline treatment at day 30 or 60 yielded very little mCherry expression (data not shown), suggesting silencing is limiting later-stage transgene induction. Timing of pathology is critical to disease modeling in neural organoids; a variety of experimental approaches suggest that later-stage organoids are more competent to harbor pathology.^21,33^ In this study, we were only able to delay mutant *MAPT* induction until day 10 of differentiation using the doxycycline inducible cassette, thus initiating pathology after the earliest stages of neuronal patterning are complete.

We also sought to understand the spatial distribution of phosphorylated tau. As expected, the neural organoids formed cortical plate-like regions composed of CTIP2+ and βIII tubulin+ neurons arranged around ventricular zone-like rosettes composed of dense nuclei (Figure 4F). Based on the rosette structures, we segmented images into ventricular-like and cortical plate-like regions and then quantified phospho-tau signal in both regions. The phospho-tau signal was mostly restricted to the cortical plate-like regions of the neural organoid tissue treated with doxycycline (97.7 ± 1.7% in cortical plate-like regions) (Figure 4G). The localization of phosphorylated tau was shifted by puromycin selection, whereby a greater proportion of positive signal was found to be localized to the ventricular-like zones (p = 0.009, two-tailed *t* test) (Figure 4G). Overall, having validated robust tau accumulation in the neural organoids due to early transgene induction, we next sought to characterize the pathological signature induced by mutant *MAPT* overexpression.

### Transcriptomic Analysis

Transcriptomic responses correlated to tau pathology have been routinely reported in the literature.^46,47^ Thus, to gain deeper insights into the cellular response elicited by inducible mutant *MAPT* overexpression, we characterized three-month-old organoids using bulk RNA sequencing after extracting high-quality RNA (RIN > 9.0, see Methods). Consistent with our protein-level characterization, *MAPT* transcripts were highly detected in doxycycline-treated organoids (Figure 5A, Supporting Table 1). Eight other transcripts were also highly upregulated (|log_2_(FC)| > 1.0): *ONECUT1, RERGL, GREB1L, AKAIN1, VGLL3, TBC1D1, FGF9*, and *CFAP61*. Of these transcripts, *MAPT*, *GREB1L*, and *TBC1D1* are expressed in excitatory neurons and display upregulation in specific populations across the course of Alzheimer’s disease (Supporting Figure 4A–B).^46^ *GREB1L* has also been associated with disrupted excitatory-to-inhibitory neural balance.^48^ Of the remaining upregulated transcripts, *ONECUT1*, *ANKAIN1*, and *VGLL3* are not typically expressed in excitatory neurons but do show upregulation in specific populations during Alzheimer’s disease, though the implications of these changes remain unelucidated (Supporting Figure 4A–B).

**Figure 5:**
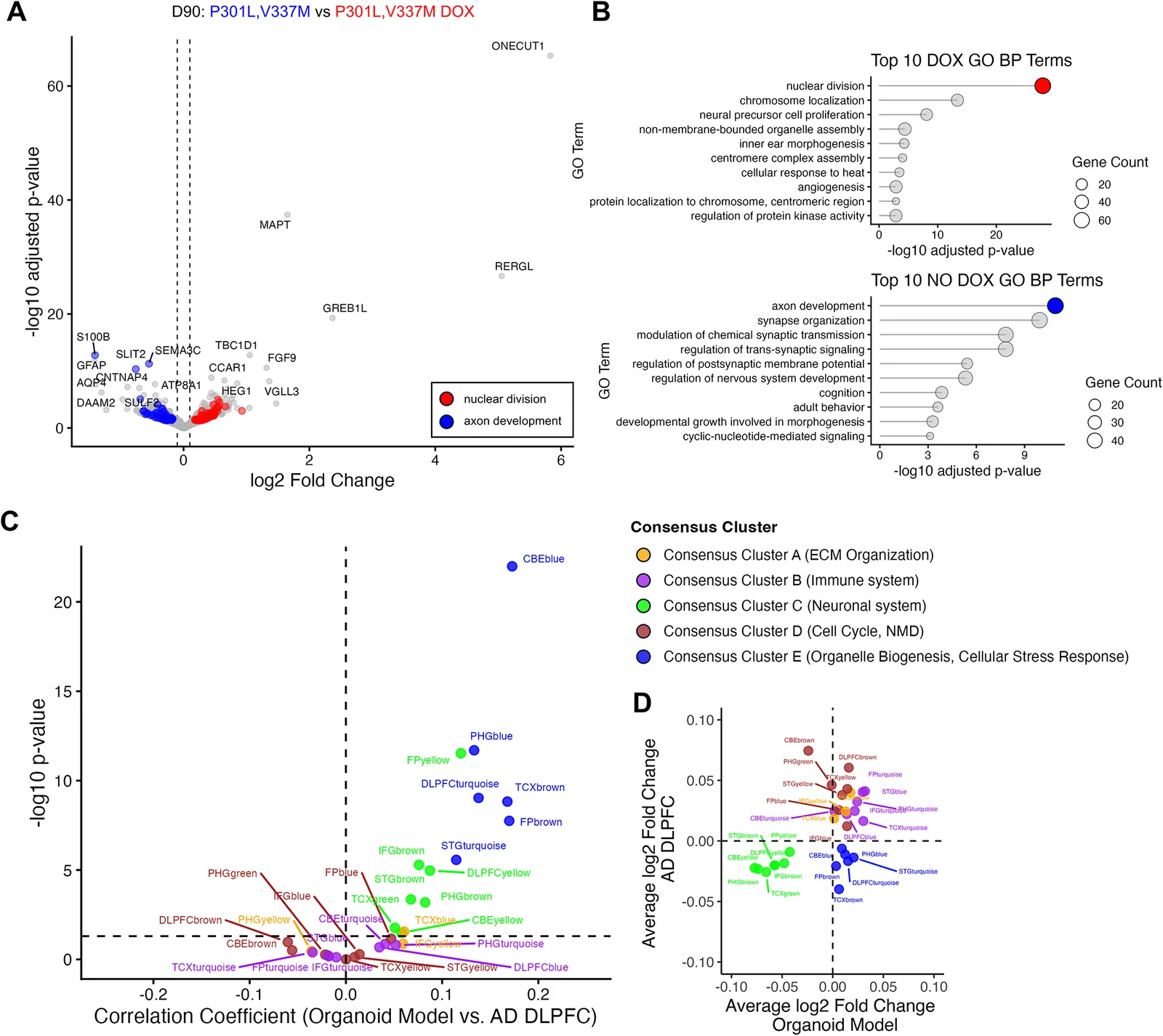
Transcriptomic response to mutant *MAPT* overexpression in neural organoids. (A) Volcano plot depicting the log_2_ fold change between engineered organoids with and without doxycycline treatment for each detected transcript (*n* = 3 individual organoids per condition, one differentiation batch). Dotted lines represent the permissive fold change cutoff of 0.1. Pathway enriched genes (pathways in panel B), labeled in red or blue, as noted in the inset legend. (B) Gene ontology enrichment plots displaying the top ten biological process (BP) gene ontology (GO) terms for each treatment group (with or without doxycycline) after list simplification to reduce redundancy (see Methods). Pathways highlighted in panel A are colored in this panel. Dot size represents number of differential genes in the set. (C) Scatter plot depicting the correlation coefficient and associated p-values of genes in each gene module between the organoid model and patient data. Each point represents a gene module, colored by biological interpretation (right). The horizontal dotted shows a cutoff of *p* = 0.05. AD DLPFC – Alzheimer’s disease dorsolateral prefrontal cortex. (D) Scatter plot depicting the average log_2_ fold change difference of transcripts per gene module. *X*-axis: fold change in the organoid model, *Y*-axis: fold change in AD DLPFC. Points are colored as in panel C.

Five transcripts were highly downregulated: *GFAP, S100B, AQP4, DAAM2*, and *TIMP4.* All five transcripts are highly expressed in glia (Supporting Figure 4A), potentially indicating a disruption of the emergence of mature glia due to mutant *MAPT* overexpression. While expression levels are initially low, some subpopulations of excitatory neurons also downregulate *S100B* and *TIMP4* across the progression of Alzheimer’s disease (Supporting Figure 4B).

Using a permissive cutoff (|log_2_(FC)| > 0.1), gene ontology analysis revealed subtle but consistent changes across organoids with mutant *MAPT* overexpression, namely, increased expression of genes related to nuclear division and neural precursor cell proliferation, and downregulation of genes related to axon development and synapse organization (Figure 5B, Supporting Tables 2–5). These expression changes would be consistent with a shift away from mature neuronal function and toward progenitor status, a process that has been noted in disease pathology.^2^ To confirm that highlighted pathways were not merely a result of thresholding choices, we also performed gene set enrichment analysis across all genes ranked by fold change, noting highly concordant results (Supporting Figure 4C, Supporting Tables 6–7).

We further sought to benchmark the measured bulk transcriptomic response in comparison to changes across Alzheimer’s disease in the human dorsolateral prefrontal cortex (DLPFC). To achieve this aim, we leveraged bulk RNA sequencing generated via the ROSMAP [Religious Orders Study and the Memory and Aging Project], MSBB [Mount Sinai School of Medicine], and Mayo studies.^49–51^ Previous analysis of this data identified 30 Alzheimer’s disease associated modules, classed into five consensus clusters based on Reactome pathway enrichment.^52,53^ In each module, we assessed correlation between transcriptomic responses to pathology in Alzheimer’s disease and perturbations in the *MAPT* overexpression organoid model.

Consensus clusters C and E displayed significant, albeit weak, positive correlation, representing concordant responses in cellular pathways of neuronal systems, organelle biogenesis, and cellular stress (Figure 5C). When additionally considering the magnitude of the average log_2_ fold change per module, consensus cluster C (Neuronal systems) displayed the clearest concordance between the organoid model and patient data (Figure 5D). No modules displayed statistically significant negative correlation. Taken together, this indicates the dominant transcriptomic signature – neuronal dysfunction and downregulation of neuron-related transcripts – accurately captures some facets of human neurodegeneration. However, the weak magnitude of correlation (scores less than 0.2 across all modules) underscores the fact that many disease processes remain unrepresented in the neural organoid model.

Dysregulation of chromatin folding resulting in differential transposable element expression has previously been reported as a facet of various tauopathies.^54–56^ We leveraged our bulk RNA sequencing data to assess whether this was the case in the mutant *MAPT* overexpressing organoids, using the TEtranscripts approach.^57^ Indeed, we detected 9 significantly upregulated and 13 significantly downregulated transposable elements, 5 of which were concordant with published analysis of Alzheimer’s disease patient tissue^55^ (Supporting Figure 5, Supporting Table 6). Taken together, this analysis suggested weak dysregulation of chromatin accessibility due to *MAPT* overexpression, albeit without statistically significant overlap with previously reported perturbations (representation factor = 1.0, *p* < 0.421, hypergeometric test).

### Functional Analysis

To characterize the functional impact of tau accumulation on neuronal function, we employed whole-cell patch-clamp electrophysiology to measure properties of single neurons at three months of culture (Figure 6). We noted that membrane properties were relatively immature across all assayed neurons. Both passive and active membrane properties were largely unchanged by the overexpression of mutant *MAPT*. We did not note differences in spontaneous excitatory postsynaptic currents, which are related to synaptic network formation.^58^ Thus, our model displays the accumulation of tau and related transcriptomic response, without substantial disruptions to functional network neuronal properties at this stage of neural organoid differentiation.

**Figure 6:**
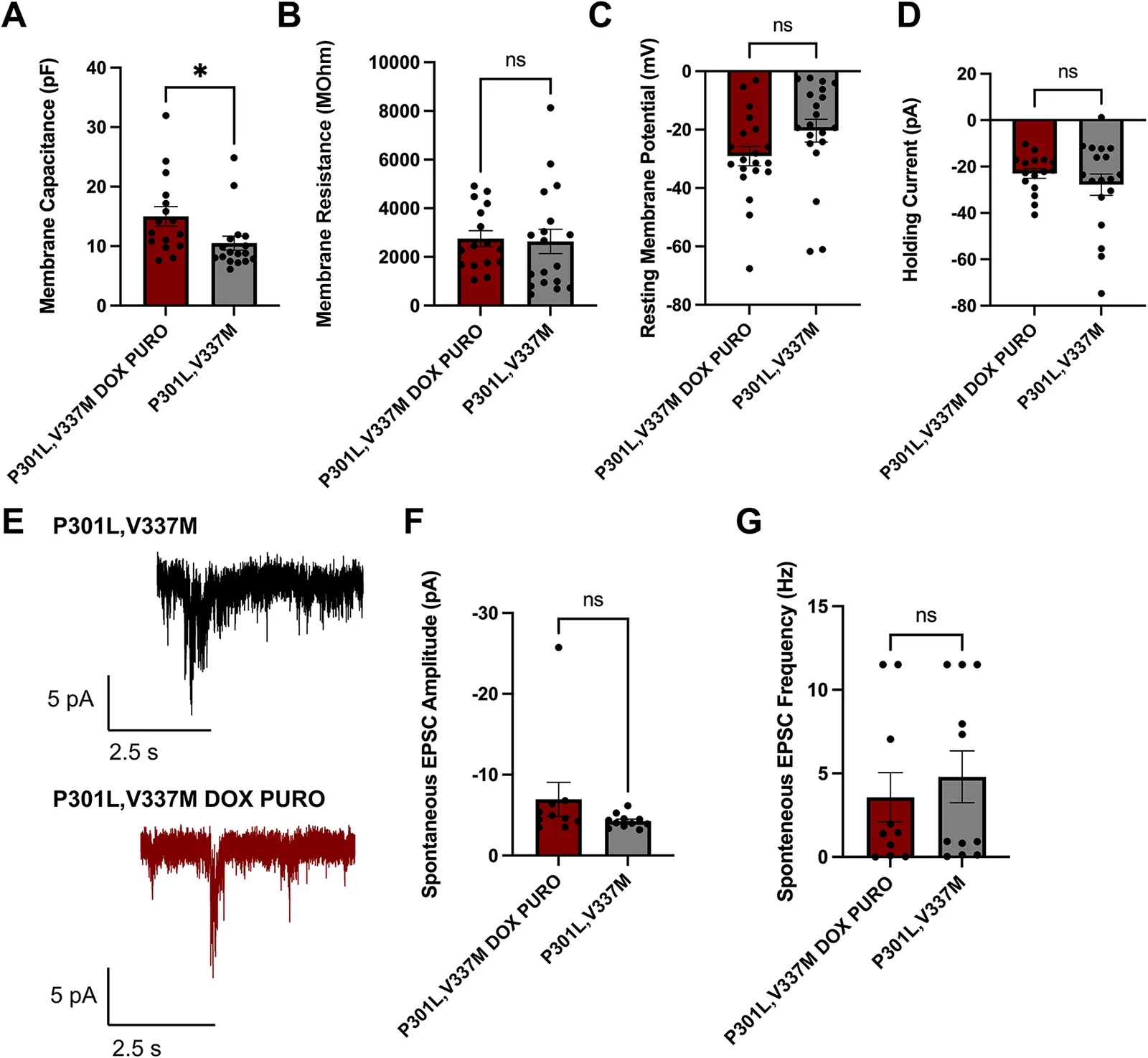
Electrophysiological characterization of neural organoids with or without mutant *MAPT* overexpression. (A–D) Patch-clamp quantification of membrane capacitance (A), membrane resistance (B), resting membrane potential (C), and holding current (D), (*n* = 16–18, individual points represent single cells, derived from *n* = 2 independent organoids per condition, one differentiation batch). *p < 0.05, n.s. – not significant, Welch’s *t* test. DOX PURO – doxycycline and puromycin addition. Bars represent the mean ± standard error of the mean. (E) Representative spontaneous excitatory postsynaptic currents (EPSC) in stimulated and nonstimulated engineered organoids. (F–G) Patch-clamp quantification of spontaneous excitatory postsynaptic current (EPSC) amplitude (F) and frequency (G), (*n* = 10–11, individual points represent single cells, derived from *n* = 2 independent organoids per condition, one differentiation batch). n.s. – not significant, Welch’s *t* test. DOX PURO – doxycycline and puromycin addition. Bars represent the mean ± standard error of the mean.

### Limitations of the Study and Future Directions

Besides progress toward full temporal control of pathology, a defined and inducible construct offers other distinct advantages that were not explored in this work: first, it could be deployed simultaneously in a diverse set of iPSC lines, thus isolating the effects of mutant *MAPT* overexpression across human genetic diversity. Second, the inducible cassette could be layered with other disease phenotypes, including amyloid β pathology. As comorbidities are extremely common in late-stage neurodegeneration, this is an important future aim.^59^ Third, this approach could also be used to control spatial pathology. By controlling which cells have the mutant cassette, for instance, via an assembloid approach, connected cultures of induced and propagated pathology could be created, representing a novel approach which has only begun to be explored in the organoid literature.^60^ Fourth, future iterations of this approach could be implemented with cell-type specific conditional expression of tau, helping to reveal cell-type specific mechanisms of neurodegenerative biology. To date, previous approaches relying on patient-derived or edited lines have lacked cell type specific control of aberrant tau expression, instead producing pathology in all cell types simultaneously that express tau or have been transfected.^21,33^

We used a dual mutant *MAPT* approach (P301L,V337M), as each individual mutation has been previously reported to produce strong phenotypes in cell cultures,^61^ but we note that patients bearing both mutations have not been reported. Thus, the dual mutant *MAPT* induction approach may not capture the exact conformation of tau aggregates that predominate in frontotemporal dementia disease cases. Moreover, the absence of immune cells (such as microglia) also limits the range of pathology displayed in the model, as it is known that immunological responses are a key player in neurodegenerative pathology.^62^ While experimentally accessible, the immature status of neurons in the neural organoid model further limits the range of pathology. Lastly, the performed electrophysiological experiments are low throughput, limiting high resolution data regarding the network connectivity of the resulting tissues.

## Methods

### Cell Culture

HEK293s were maintained on tissue culture plastic in DMEM/F12 (Corning) supplemented with 10% fetal bovine serum (qualified, US origin, Fisher Scientific), 6 mM GlutaMAX supplement (Thermo Fisher Scientific), and 1:100 v/v Pen-Strep (Thermo Fisher Scientific). Upon reaching ∼ 70% confluence, HEKs were passaged using TrypLE (Gibco).

Induced pluripotent stem cells (iPSCs) (reference line KOLF2.1J^42^) were obtained from Jackson Laboratory and cultured in StemFlex (ThermoFisher, A3349401) on 6-well plates coated with Matrigel (Fisher, 354230). Upon reaching ∼ 70% confluence, iPSCs were passaged using Versene (Gibco, 15040066).

Neural organoids were generated using the STEMdiff Cerebral Organoid Kit (#08570) with modifications from the manufacturer’s protocol. After dissociation by Accutase (Gibco, A1110501), iPSC aggregation was achieved in an ultralow attachment 96-well plate (S-Bio, MS-9096UZ) with approximately 9000 cells per well. On days 2–7, the protocol was supplemented with dual-SMAD inhibitors SB43152 (10 μM, Tocris 16-141-0) and LDN193189 dihydrochloride (2.5 μM, Tocris 60-531-0). Organoids were not embedded in extracellular matrix during the experiment. At day 30, organoids were transferred to an ultralow attachment 48-well plate (Greiner, 677970) for culture on an orbital shaker at 100 rpm.

For experiments including puromycin during organoid culture, 0.1 μg/mL puromycin was supplemented into all culture media from day 5 onward. For experiments including doxycycline during organoid culture, 2 μg/mL doxycycline was supplemented into all culture media from day 10 onward. Monolayer experiments in HEK293 and KOLF2.1J iPSC cultures also employed a doxycycline concentration of 2 μg/mL.

### Cloning

The mutant *MAPT* plasmid was designed in SnapGene software (v8.0.2) and assembled using the NEBuilder HiFI DNA assembly mix. The plasmid is based on a previously designed Sleeping Beauty construct (Addgene #60495, a kind gift from Eric Kowarz)^63^ and a mutant *MAPT* construct (Addgene #176267, a kind gift from Tsuneya Ikezu).^64^ The final plasmid consists of the following elements: ampicillin resistance for bacterial colony selection, puromycin resistance for iPSC clone selection, and the Tet-On system, which controls the expression of *MAPT* and mCherry, separated by the P2A cleavage site. After assembly, the plasmid was transformed into *E. coli* and plated on ampicillin supplemented agar plates. The next day, overnight liquid broth cultures in LB supplemented with ampicillin were generated from picked colonies, and the plasmid was extracted using a miniprep DNA purification kit (Qiagen). As the source plasmid for *MAPT* contained the single P301L point mutation, the QuikChange XL Site-Directed Mutagenesis Kit (Agilent, 200517) was used based on manufacturer specifications to layer in the V337 M mutation. Sanger sequencing was used to verify the construct.

### Transfections

Transfection was carried out with two plasmids: the donor plasmid as described above, and a Sleeping Beauty transposase plasmid (Addgene 34879, a kind gift from Zsuzsanna Izsvak).^39^ Plasmids were mixed in 200 μL of Opti-MEM (Gibco) at a 1:1 mass ratio (1 μg of each plasmid). Afterward, 6 μL of room-temperature TransIT-LT1 Reagent (Mirus) was added. The complexes were incubated for 15–20 min at room temperature. For HEK293 transfections, plasmid complexes were added directly to 50% confluent cells in a 12-well plate. For iPSC transfections, cells were passaged and then seeded at a controlled density in well plates already containing the plasmid complexes. A density of 100,000 cells per well of a 12-well plate was used, and cells were seeded in StemFlex media (Gibco) supplemented with ROCK inhibitor (10 μM Y-27632, Tocris). Cells positive for constructs were selected first by puromycin addition (1 μg/mL for HEK293 cells, 0.33 μg/mL for iPSCs). Copy number or genomic integration sites were not assayed.

### Fluorescence-Activated Cell Sorting

The main iPSC line used in this study was further purified by fluorescence-activated cell sorting (FACS). After 24 h of doxycycline treatment (1 μg/mL), cells were dissociated with a 5 min incubation in Accutase (Stem Cell Technologies). After pelleting and resuspension in flow sorting buffer (5% fetal bovine serum, 95% DPBS), cells were strained and sorted based on mCherry expression levels (detection in the 555 channel) using a FACSDiva 8.0.2 instrument. Cells with high mCherry levels were bulk sorted into StemFlex supplemented with ROCK inhibitor (10 μM Y-27632, Tocris) and Antibiotic-Antimycotic (Gibco, 15240062). Surviving iPSC colonies were expanded before further use.

### Flow Cytometry

For analytical flow experiments, cells were digested into a single-cell suspension by a 3 min incubation in TrypLE (Gibco) and pelleted. Cells were then resuspended in 2% paraformaldehyde (ThermoFisher #J19943-K2, diluted with distilled water) for 5 min before repelleting. A subsequent wash with DPBS supplemented with 0.05% bovine serum albumin (BSA) was performed before final resuspension in DPBS supplemented with 0.05% BSA. Analyses were then performed on a BD Fortessa 5 Laser flow cytometer. 20,000 single cell events were recorded per sample.

#### Gating of HEK293 Experiments

Cells were gated to exclude doublets and debris, and then mCherry+ was defined at a threshold giving a 2% background activation rate in the unstimulated condition (Supporting Figure 6A). No unengineered cells (true mCherry- controls) were utilized.

#### Gating of iPSC Experiments

Cells were gated to exclude doublets and debris, and then mCherry+ was defined at a threshold giving a 0% background activation rate in unengineered control conditions (Supporting Figure 6B).

### Live Imaging

Cells were live imaged routinely with a Leica DM8 epifluorescence microscope to assess mCherry expression levels and were quantified in ImageJ (see Image Quantification).

### Protein Extraction and Western Blotting

To collect protein lysates from HEK cells, cells were washed with DPBS and then lifted using a sterile cell scraper (Cell Treat). The collected material was pelleted via centrifugation (∼150*g* for 5 min), the supernatant was aspirated, and the pellet was frozen overnight at −20 °C. The next day, pellets were resuspended in DPBS, 0.05% Triton X-100, 1% v/v protease inhibitor cocktail (Sigma-Aldrich #P8340), and 1% v/v phosphatase inhibitor cocktail (Sigma-Aldrich #P0044). After trituration, samples were incubated at 4 °C for 10 min, spun down at 500*g* for 5 min, and then immediately spun down further at 1000*g* for 5 min. The supernatant was kept and assayed for protein concentration using a standard Pierce BCA protein assay kit (Thermo Scientific #23225).

To collect protein lysates from organoids, organoids were removed from their cell culture media and rapidly frozen on dry ice. RIPA buffer (Thermo Fisher Scientific #89900) supplemented with protease inhibitor cocktail (Sigma-Aldrich #P8340) and phosphatase inhibitor cocktail (Sigma-Aldrich #P0044) was then added to the tubes containing organoids and allowed to sit on ice for 20 min. Organoids were then manually disrupted using a P200 pipet tip, and sonicated at 20% power, 10 s on, 10 s off for three cycles. Lastly, the homogenized material was centrifuged at 14,000*g* at 4 °C for 10 min and the supernatant was collected. Protein concentration was assayed using a standard Pierce BCA protein assay kit (Thermo Scientific #23225).

For Western blots, a 7.5% TGX gel (BioRad #5671025) was utilized. Proteins were prepared in Laemmli buffer (BioRad #1610747), supplemented with β-mercaptoethanol (Sigma #M6250-10 ML), and boiled at 95 °C for 5 min. After cooling for 5 min on ice, 20 μg of protein was loaded per sample, based on the results of the BCA protein assay. The electrophoresis apparatus was run at 80 V until completion, with Tris-based running buffer including SDS (KD Medical #50-101-8898). The Trans-Blot Turbo Transfer System was used to transfer gel-bound proteins to a nitrocellulose membrane. After 30 min of incubation with Li-Cor Intercept (TBS) Blocking Buffer (Fisher #NC1660550), primary antibodies suspended in blocking buffer were added overnight and allowed to incubate at 4 °C. The next day, after three 5 min washes with Tris buffered saline (ThermoFisher #J60764.K2) supplemented with Tween 20 (Sigma-Aldrich #P9416) (TBST), secondary antibodies diluted in TBST were added and then incubated at room temperature for between 1.5–3 h. After three additional 5 min TBST wash steps, the finalized membrane was imaged at a Li-Cor Odyssey XF imaging system.

### Monolayer Fixing and Immunofluorescent Labeling

Monolayer cultures were fixed in 4% paraformaldehyde (ThermoFisher #J19943-K2) for 15 min at room temperature. Next, they were washed three times with DPBS, 10 min each at room temperature. Cultures were then incubated at 4 °C in blocking buffer (5% v/v goat serum, Life Technologies; 0.3% v/v Triton X-100, Sigma) for 1 h. Overnight, cultures were incubated in primary antibodies at 4 °C. The next day, after three 10 min washes at room temperature with DPBS, cultures were incubated in secondary antibodies for an additional 3 h at 4 °C. After three 10 min washes at room temperature with DPBS, cultures were imaged in DPBS. Epifluorescence images were acquired with a Leica DM8 epifluorescence microscope.

### Organoid Fixing, Cryosectioning, and Immunofluorescent Labeling

Organoids were fixed in 4% paraformaldehyde (ThermoFisher #J19943-K2) overnight at 4 °C. The next day, after three washes in PBS, 15% sucrose (ThermoFisher #J65148.36) was added. Organoids were incubated overnight at 4 °C to allow equilibration and full penetration. The next day, 30% sucrose was added. Organoids were again incubated at 4 °C to allow equilibration and full penetration. A 7.5% gelatin (ThermoFisher #G1890)/10% sucrose solution was prepared and warmed to 37 °C; organoids were then embedded in cryo-molds surrounded completely by the gelatin/sucrose solution. After polymerization at 4 °C for 15–20 min (to allow setting of the gelatin), the embedded organoids were flash frozen (1–2 min) in an isopentane (ThermoFisher #126470010) dry ice bath between −50 and −30 °C. After flash freezing, samples were stored at −80 °C, sectioned on a cryostat, and mounted on slides.

For immunofluorescent labeling of sectioned slides, slides were first allowed to thaw to room temperature and then were washed three times with PBS. Tissues were then permeabilized for 10 min with 0.2% Tween 20 at room temperature. After three PBS washes, tissues were then blocked using 5% BSA (Research Products International #9048-46-8) in PBS at room temperature for 2 h. Primary antibodies were applied overnight at 4 °C in PBS containing 1% BSA. The next day, secondary antibodies diluted in PBS containing 1% BSA were applied for 3 h at room temperature, followed by three PBS washes. Finally, Prolong Gold with DAPI (Thermo Fisher #P36931) was applied, coverslips were mounted, and nail polish was used to seal the slide. Slides were imaged on a Leica DM8 epifluorescence microscope. No antibodies were employed to visualize mCherry, as fluorescence signal was retained after fixation.

### Confocal Imaging

For high resolution imaging, slides were imaged at 100X on a Nikon spinning disk confocal with super resolution by optical pixel reassignment (SoRa). Images were deconvolved using a Richardson-Lucy algorithm of ten iterations.

### Bulk RNA Sequencing

RNA was extracted from organoids using the RNeasy Plus Mini Kit (Qiagen #74134), following manufacturer’s specifications. To homogenize tissue, stainless steel beads (Qiagen #69989) and 3 min at 3000 rpm in a TissueLyser (Qiagen) homogenizer were employed. The final RNA product was eluted in RNase free water and then assessed for quality using an RNA ScreenTape. Samples exceeding a RIN of 9.0 were used (mean: 9.36, standard deviation: 0.15). Standard mRNA enrichment, cDNA library preparation (Stranded mRNA Library Prep, NEB), and sequencing of paired-end reads on an Illumina NovaSeq XP were then performed, targeting 50 M reads per sample.

### Bulk RNA Sequencing Data Analysis

Paired end reads were aligned to the human genome (hg38) using Rsubread (v.2.18.0).^65^ Differentially expressed genes were then computed using DESeq2 (v.1.44.0).^66^ Transcripts with an adjusted *p* value <0.05 and an absolute log_2_ fold change >0.1 were then analyzed via gene ontology analysis. The clusterProfiler package (v.4.12.6)^67^ was used with default settings and resulting biological process lists were simplified using the simplify function with a cutoff of 0.5. To perform gene set enrichment analysis, all transcripts were rank ordered by log_2_ fold change and then analyzed using the WebGestalt tool for nonredundant biological pathways.

To compare with patient data, a gene module correlative approach was utilized, as previously described.^53^ Human coexpression module data were obtained from the Synapse data repository (syn11932957). Differential expression analysis (log fold changes) derived from the ROSMAP [Religious Orders Study and the Memory and Aging Project], MSBB [Mount Sinai School of Medicine], and MayoRNaseq studies for the dorsolateral prefrontal cortex were obtained from the Synapse data repository (syn14237651).^49–51^ Log fold changes modeled on harmonized diagnosis status were utilized. After filtering to retain transcripts in common between organoid and patient data sets, a correlation coefficient was computed for each human coexpression module using the built-in R cor.test function. Average log_2_ fold change per data set per module was also computed.

Transposable element annotation was performed using the atena package (v.1.10.0).^68^ In brief, RepeatMasker annotations for hg38 were downloaded from UCSC and then used in the TEtranscripts approach.^57^ After annotating LINE, SINE, LTR, DNA, and other transposable elements per sample, DESeq2 was used to compare expression levels between samples. Transcripts with an absolute log_2_ fold change >1 and an adjusted p value <0.05 were considered significantly different between conditions. Fold change and adjusted p values were then compared directly with the data presented by Grundman et al., generated from post-mortem human tissue.^55^

### Electrophysiological Characterization

Electrophysiological characterization was performed as previously described.^69^ Whole-cell recordings were performed after transferring organoids to artificial cerebral spinal fluid (aCSF, in mM: 119 NaCl, 2.5 KCl, 1.3 MgCl2–6H2O, 2.5 CaCl2–2H2O, 1.0 NaH2PO4-H2O, 26.2 NaHCO3, and 11 glucose; 290–295 mOsm) at room temperature and patched with 4–6 MΩ recording pipettes (P1000 Micropipette Puller) filled with a potassium-gluconate based internal solution (in mM: 135 K-gluconate, 5 NaCl, 2 MgCl2, 10 HEPES, 0.2 EGTA, 2.5 Mg2-ATP, 0.2 Na2-GTP). Spontaneous excitatory postsynaptic currents (sEPSCs) were collected while holding cells at −70 mV in the voltage-clamp configuration. Inclusion criteria for cells were an access resistance of ≤ 25 mOhms, holding current <−100 pA at −70 mV, and no change in either metric >20%. Recordings were low-pass filtered at 2 kHz and digitized at 20 kHz using a DigiData 1550A (Molecular Devices). Data collected from electrophysiology experiments were initially analyzed in Clampfit 10.7 and then graphed using GraphPad Prism 10.

### Image Quantification

Immunofluorescent images were analyzed in ImageJ (v1.53t).^70^ For monolayer cultures, mean fluorescence intensity (MFI) was computed across entire images, as cell confluency was relatively similar. Total organoid area was estimated by manual tracing, based on the DAPI channel. Percent area (mCherry or p-tau) was estimated by thresholding and binary segmentation of the respective channel, and then normalization to the total organoid area. Thresholding for “high intensity tau” (Figures 3 and 4) was defined manually as the lowest threshold producing values of zero in nontreated controls, thus excluding endogenous tau expression. Imaging and thresholding parameters were held constant across all relevant comparisons. Segmentation between ventricular-like and cortical plate-like regions was done by hand, based on cellular morphology. For western blot analysis, regions of interest were drawn in ImageJ using the protein ladder and then densitometry analyses were performed. All values were strictly normalized to loading controls to control for potential differences in loading.

### Statistical Analyses

Conventional statistical analyses on data produced by live imaging, immunofluorescent staining, western blot, and patch-clamping were performed in GraphPad Prism 10. All data were tested via a Shapiro-Wilk test for normality. For comparisons between two samples that fit normal distributions, unpaired *t* tests were used if assuming equal variance (homoscedasticity as assessed by an F-test to compare variances), otherwise, Welch’s *t* tests were employed. Nonparametric Mann–Whitney tests were employed for comparisons between non-normal sample distributions. For multiple conditions where all conditions fit a normal distribution, ordinary one-way ANOVA and Tukey’s multiple comparisons tests were employed to make comparisons between all groups. Otherwise, Dunnett’s multiple comparisons tests were employed to compare all groups to the control only. For data sets where any conditions did not fit a normal distribution, a Kruskal–Wallis test was instead employed. For data sets with three or fewer data points per condition, we assumed the distribution was normal, as normality testing is underpowered.

### Key Resources

The key resources are listed in Table 1.

**Table 1:** 

REAGENT or RESOURCE	SOURCE	IDENTIFIER
Antibodies		
Rat monoclonal [25B6] anti-CTIP2, 1:250 (thin-sections)	Abcam	Cat#18465; RRID: AB_2064130
Mouse tau monoclonal (TAU-5), 1:1000 (western blot)	ThermoFisher	Cat#AHB0042; RRID: AB_2536235
Mouse cyclophilin B (PPIB) [CL3901], 1:1000 to 1:500 (western blot)	Abcam	Cat#236760; RRID: AB_2787028
Rabbit monoclonal phosphorylated tau (pS404) [EPR2605], 1:1000 (western blot), 1:500 (thin-sections)	Abcam	Cat#92676; RRID: AB_10561457
Mouse β III tubulin [2G10], 1:500 (thin-sections)	Abcam	Cat#78078; RRID: AB_2256751
Mouse monoclonal phosphorylated tau (pS202, pT205) AT8, 1:1000 (western blot)	ThermoFisher	Cat#MN1020; RRID: AB_223647
Rabbit polyclonal oligomeric tau T22, 1:1000 (western blot)	Millipore	Cat#ABN454; RRID: AB_2888681
Cell lines		
HEK293		
KOLF iPSCs	The Jackson Laboratory	
Deposited Data		
Raw and processed bulk RNA sequencing	This paper	Array Express: E-MTAB-16510
Alzheimer’s coexpression modules	Wan et al.^52^	Synapse: syn11932957
Alzheimer’s disease bulk RNA sequencing differential expression	De Jager et al., Wang et al., Allen et al.^49–51^	Synapse: syn14237651
Alzheimer’s disease temporal cortex bulk RNA sequencing	Grundman et al.^55^	

## Supplementary Material





## Data Availability

Bulk RNA sequencing data are deposited at array express under accession code E-MTAB-16510. Code to reproduce the derived figures can be accessed via github: andrewkjar/dual_mutant_MAPT_brain_organoid.

## Notes

*MAPT* organoid (XLSX)
Sanger sequencing and plasmid design of a P301L, V337M *MAPT* inducible cassette; thin-slice imaging of overexpressed *MAPT* in neural organoids; high-resolution imaging of overexpressed *MAPT* in neural organoids; differentially expressed transcript dynamics in patient tissue; analysis of transposable elements; and gating strategy for analytical flow experiments (PDF)
