# Supplementary material for: Conditional Mutant MAPT Overexpression in Neural Organoids Elicits Robust Tau Accumulation and Pathological Transcriptomic Responses
Source: ACS Chem Neurosci. 2026 Sep 28;17(21):3501–16. doi: 10.1021/acschemneuro.6c00376 (PMC13741792; doi:10.1021/acschemneuro.6c00376)
Supplement: Supplementary file 1 [file cn6c00376_si_002.pdf]

Supporting Information for Publication

Conditional Mutant *MAPT* Overexpression in Neural Organoids Elicits Robust Tau Accumulation and Pathological Transcriptomic Responses

Andrew Kjar[1], Hyosung Kim[2], Madeline R. Spetz[1], Zev Jarrett[3], Melanie Fernandez[1], Jaime Wang[1], Maria L. Russotti[1], Caroline Bodnya[4], Daniel Chavarria[2], Timothy J. Hohman[5], Vivian Gama[4,6,7], Brad Grueter[3,8,9], Jonathan M. Brunger[1,6,10], Ethan S. Lippmann[1,2,6,*]

[1]Department of Biomedical Engineering, Vanderbilt University, Nashville, TN, USA

[2]Department of Chemical and Biomolecular Engineering, Vanderbilt University, Nashville, TN, USA

[3]Department of Pharmacology, Vanderbilt University, Nashville, TN, USA

[4]Department of Cell and Developmental Biology, Vanderbilt University School of Medicine, Nashville, TN, USA

[5]Vanderbilt Memory and Alzheimer's Center, Vanderbilt University Medical Center, Nashville, TN, USA

[6]Vanderbilt Brain Institute, Vanderbilt University, Nashville, TN, USA

[7]Vanderbilt Center for Stem Cell Biology, Vanderbilt University, Nashville, TN, USA

[8]Department of Anesthesiology, Vanderbilt University Medical Center, Nashville, TN, USA

[9]Vanderbilt Center for Addiction Research, Vanderbilt University, Nashville, TN, USA

[10]Vanderbilt-Ingram Cancer Center, Vanderbilt University Medical Center, Nashville, TN, USA

*Lead contact (ethan.s.lippmann@vanderbilt.edu)

A *Sanger sequencing to confirm G → A at 337 after QuikChange cloning*

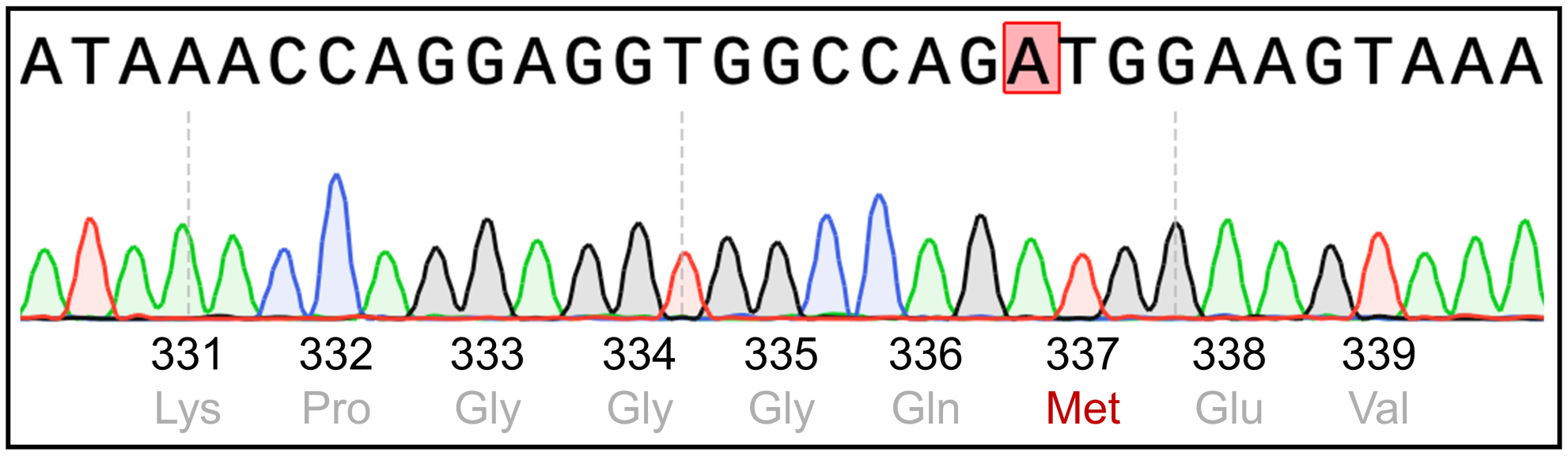

B *Full plasmid design*

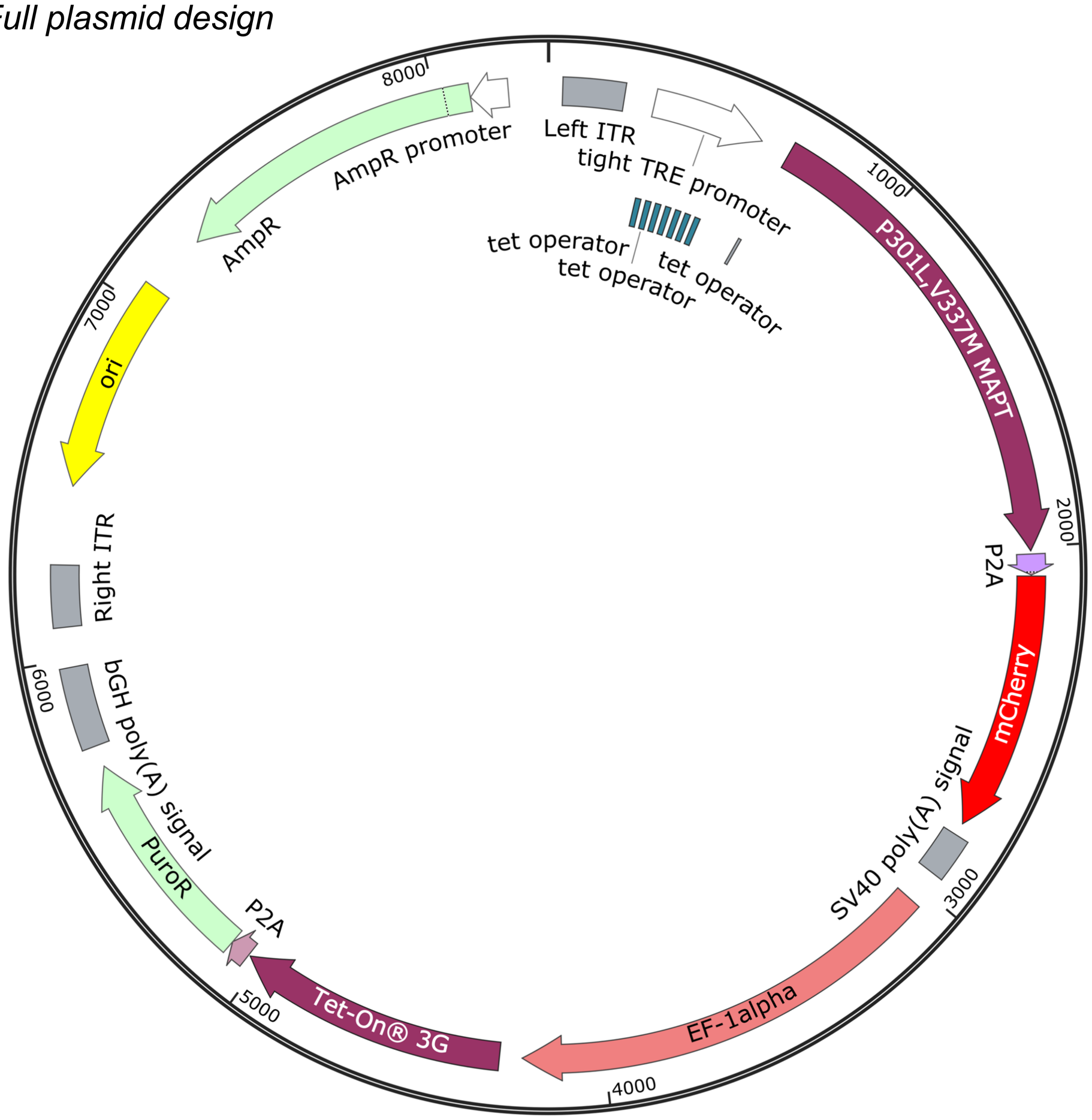

Supplemental Figure 1. Sanger sequencing and plasmid design of a P301L,V337M *MAPT* inducible cassette.

(A) Sanger sequencing demonstrating the layered V337M point mutation in the *MAPT* transcript. The parent sequence contained the P301L mutation.

(B) Full plasmid map.

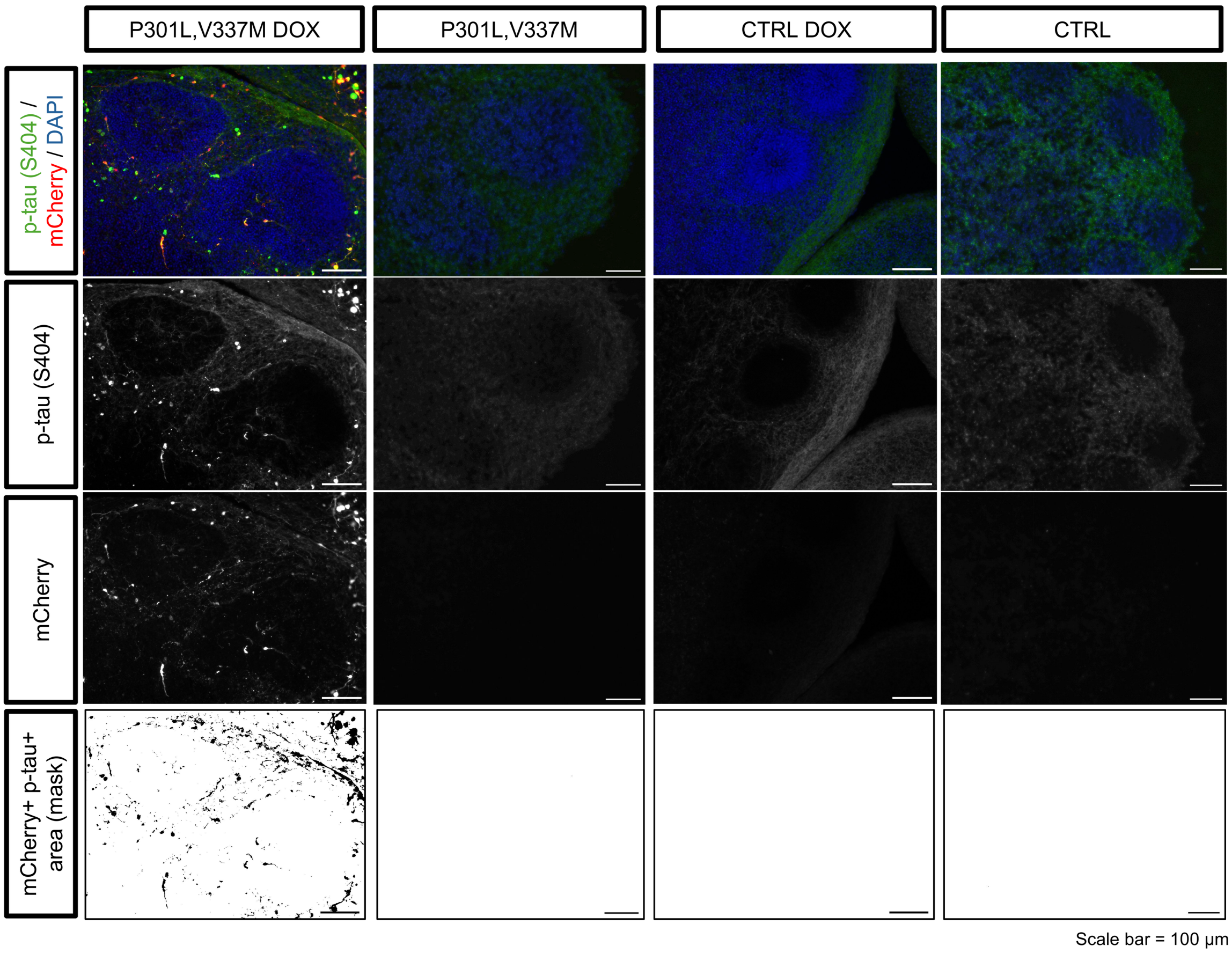

Supplemental Figure 2. Thin-slice imaging of overexpressed *MAPT* in neural organoids.

Representative immunofluorescent imaging of thin-slice sections of doxycycline-treated engineered organoids (P301L,V337M DOX) and comparison conditions: engineered organoids - P301L,V337M, doxycycline-treated control organoids - CTRL DOX, and control organoids - CTRL. Visualizing mCherry and pS404. A binary mask of mCherry+ p-tau (S404)+ area is displayed on the final row, to aid visualization of colocalization. Scale bar = 100 µm.

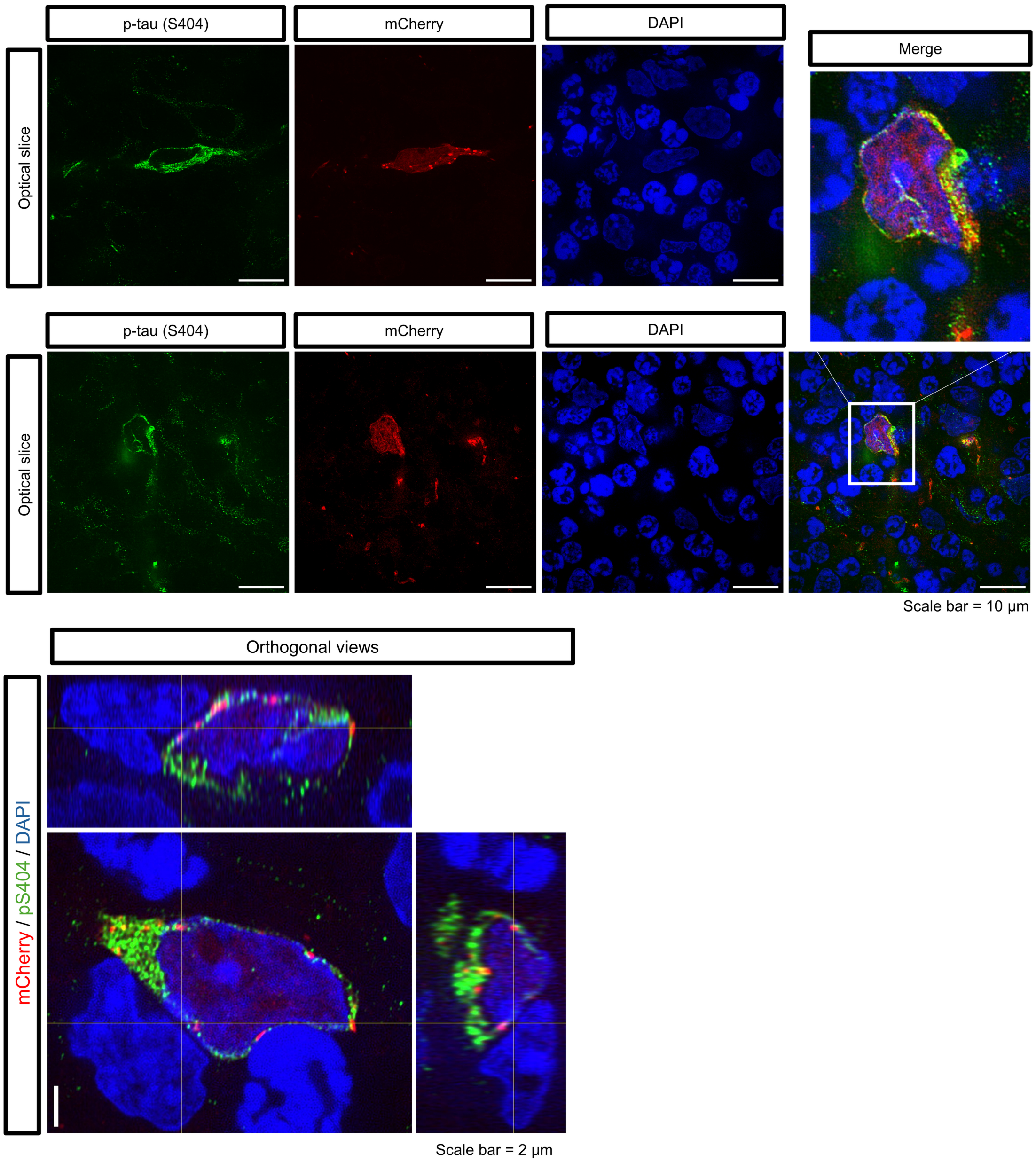

Supplemental Figure 3. High resolution imaging of overexpressed *MAPT* in neural organoids.

Additional high-resolution confocal imaging of thin-slice section of doxycycline-treated engineered organoids, visualizing mCherry, pS404, and DAPI. Scale bars = 10 µm or 2 µm, as noted. Images are noted as optical slices or orthogonal views of three-dimensional reconstruction.

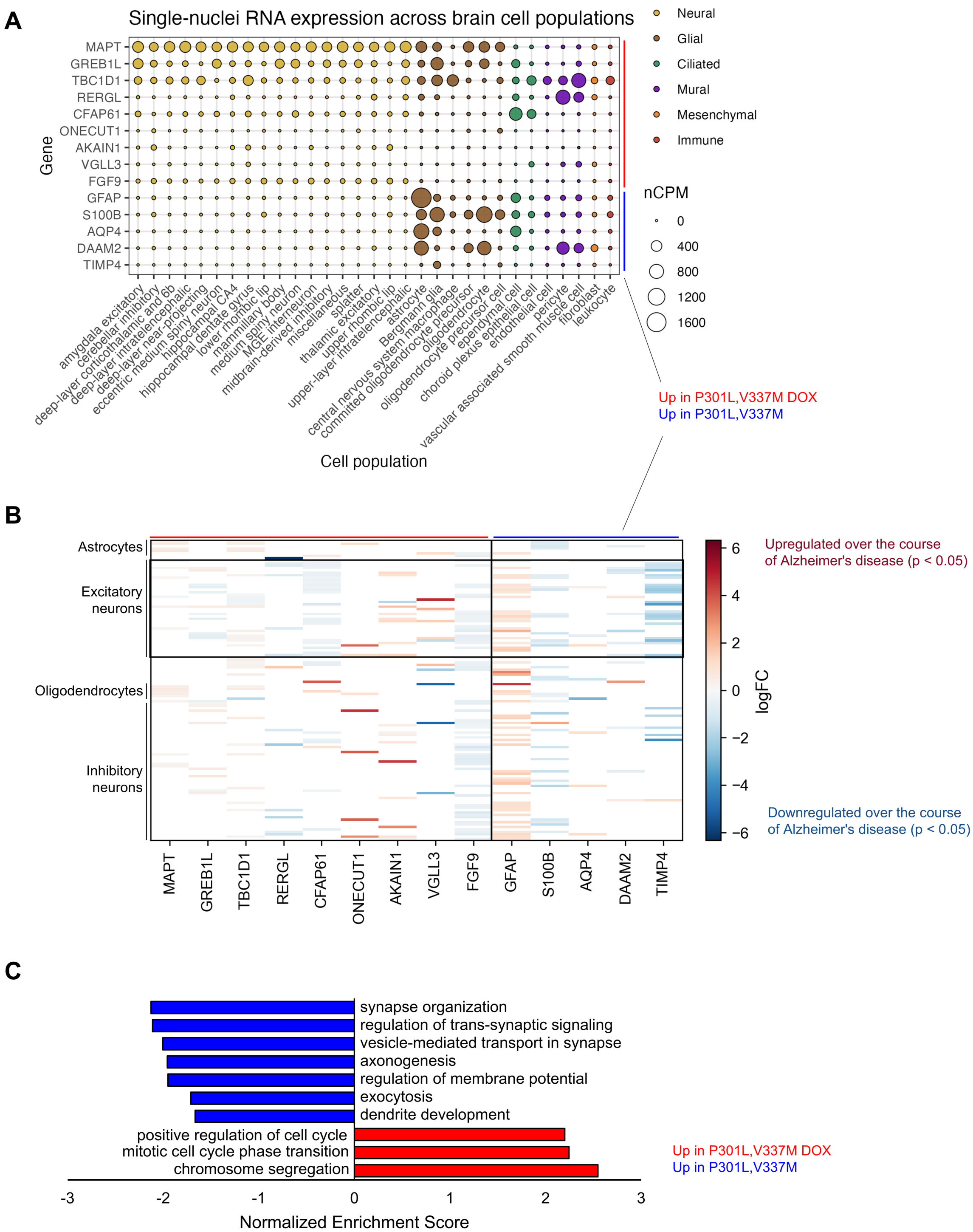

Supplemental Figure 4. Differentially expressed transcripts dynamics in patient tissue.

(A) Expression patterns of top differentially expressed transcripts from Figure 5A. Plotted from the Human Protein Atlas brain single-nuclei RNA sequencing dataset, published by Siletti et al. (2023).

(B) Differential expression of transcripts over the course of Alzheimer's disease. Rows represent specific cell subpopulations; major blocks of related cell types are noted on the left. Columns represent each transcript from panel A. Color represents log fold change across Alzheimer's disease progression, red – upregulated, blue – downregulated, white – not significant ($p > 0.05$). Differential expression over disease progression computed by Gabitto et al. (2024).

(C) Gene set enrichment analysis (GSEA) of differential transcripts between engineered organoids with and without doxycycline treatment. All top GSEA pathways are shown, after weighted cover reduction and filtration to a false discovery rate < 0.05.

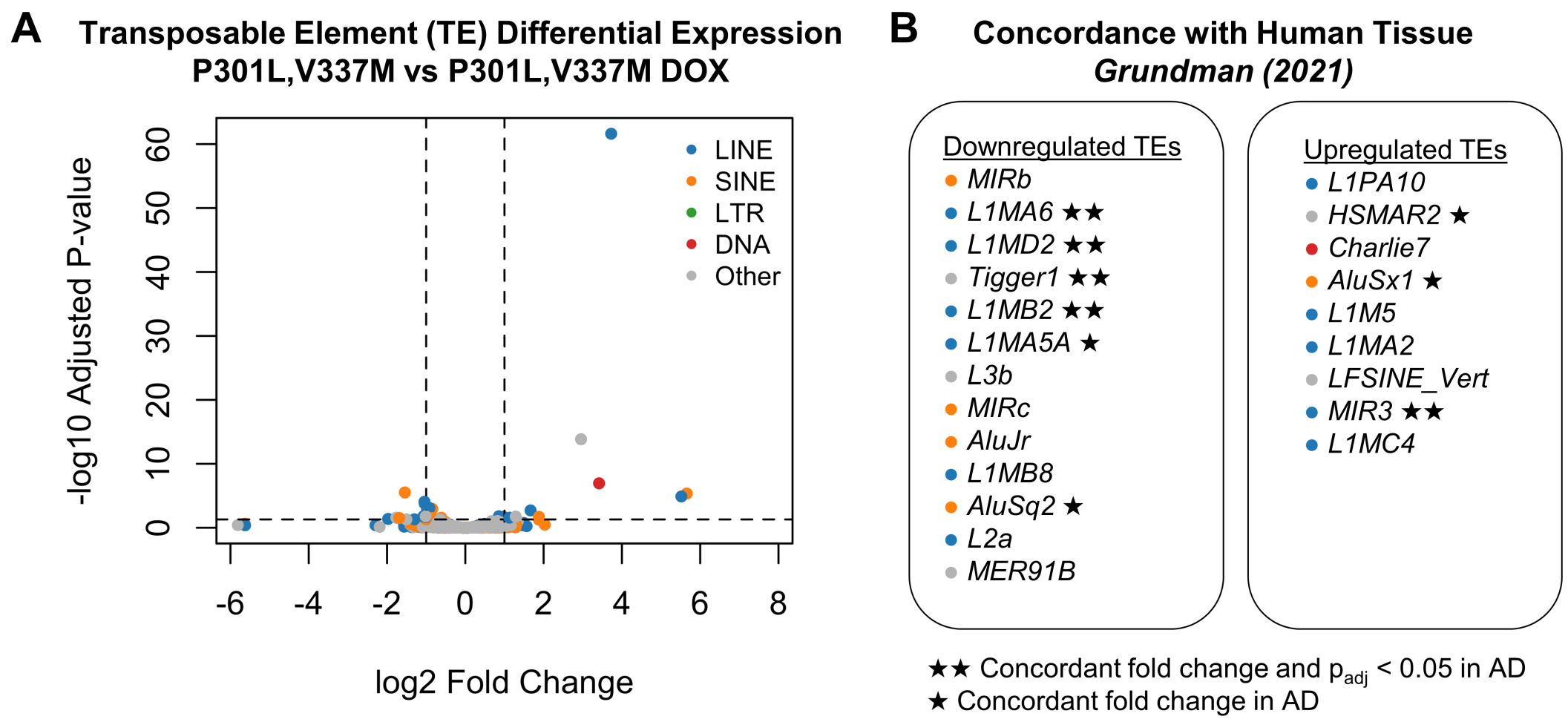

Supplemental Figure 5. Analysis of transposable elements.

(A) Volcano plot depicting the $\log_2$ fold change between engineered organoids with and without doxycycline treatment for each detected transposable element. Dotted lines represent the fold change cutoff of 1. Transcripts are colored by family.

(B) Lists of downregulated and upregulated transposable elements, colored by family as in panel A. Stars indicate concordance with data from Alzheimer's disease patients from Grundman et al. (2021). One star – concordant fold change sign; two stars – concordant fold change sign and reaching statistical significance in both datasets.

A HEK flow cytometry gating

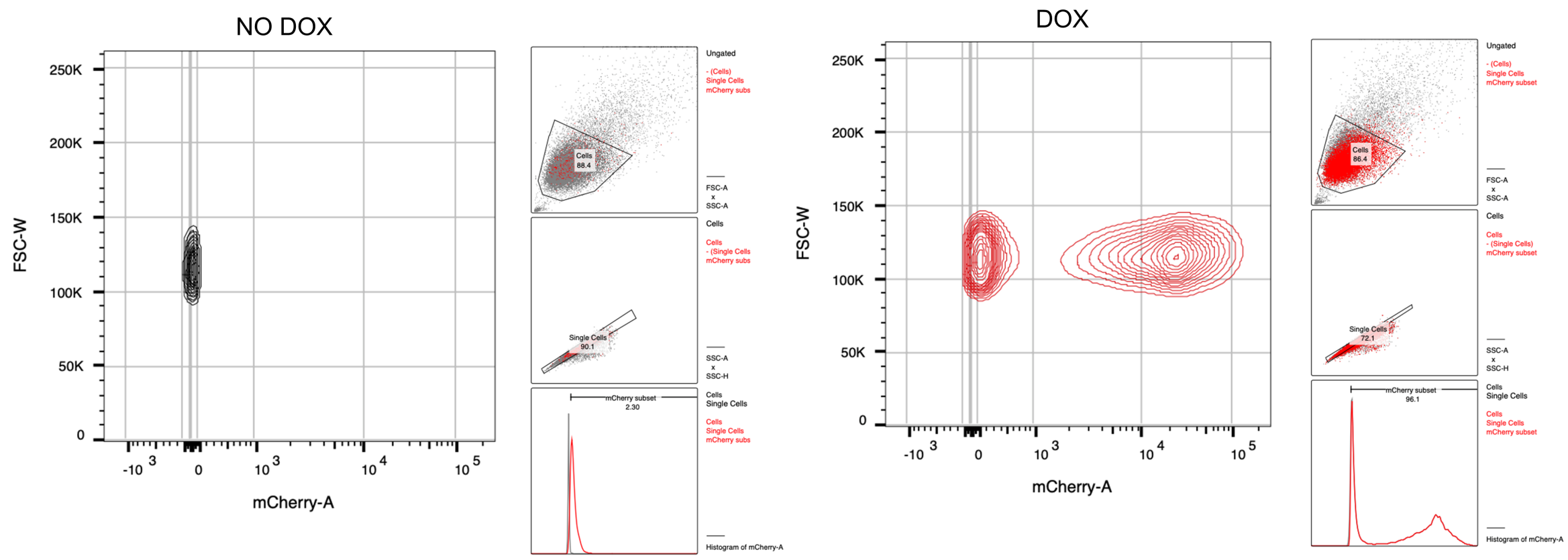

B iPSC flow cytometry gating

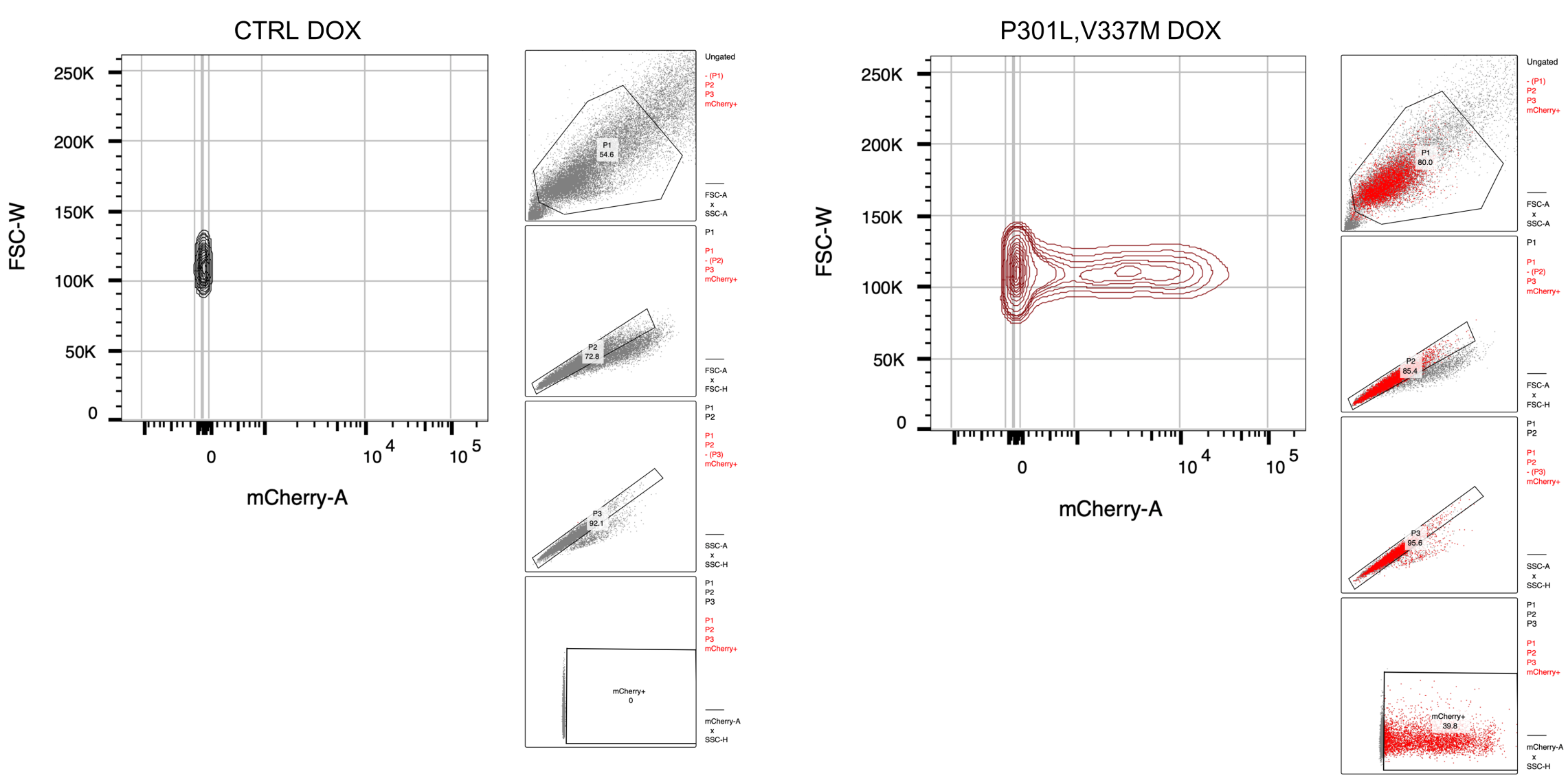

Supplemental Figure 6. Gating strategy for analytical flow experiments.

(A) Gating strategy for HEK293 experiments (presented in Figure 1). Back-gating plots display the following from top to bottom: forward scatter by side scatter (FSC-A x SSC-A), side scatter by side scatter (SSC-A x SSC-H), and a histogram of mCherry-A. The mCherry+ subset designation is shown on the final histogram. DOX – doxycycline.

(B) Gating strategy for iPSC experiments (presented in Figure 2). Back-gating plots display the following from top to bottom: forward scatter by side scatter (FSC-A x SSC-A), forward scatter by forward scatter (FSC-A x FSC-H), side scatter by side scatter (SSC-A x SSC-H), and mCherry-A by side scatter (mCherry-A x SSC-H). The mCherry+ subset designation is shown on the final scatter plot. CTRL – control, un-engineered KOLF2.1J line; P301L,V337M – engineered line; DOX – doxycycline.
